# Correction to: Quantitative genetics of microbiome-mediated traits

**DOI:** 10.1093/evolut/qpag092

**Published:** 2026-06-04

This is a correction to: Bob Week, Peter L Ralph, Hannah F Tavalire, William A Cresko, Brendan J M Bohannan, Quantitative genetics of microbiome-mediated traits, *Evolution*, Volume 79, Issue 11, November 2025, Pages 2487–2502, https://doi.org/10.1093/evolut/qpaf171. 

In the originally published version of this manuscript, the two panels within Figure 2 were inadvertently switched.

The figure has been corrected to re-position the panels.

