# Supplementary material for: Quantitative genetics of microbiome-mediated traits
Source: Evolution. 2025 Aug 29;79(11):2487–502. doi: 10.1093/evolut/qpaf171 (PMC12620654; doi:10.1093/evolut/qpaf171)
Supplement: qpaf171_Supplemental_Files [file qpaf171_supplemental_files.zip › QGMMT-SUPP-FINAL.pdf]

Supplementary Material for

Quantitative Genetics of Microbiome Mediated Traits

Bob Week, Peter L. Ralph, Hannah F. Tavalire, William A. Cresko, Brendan J. M. Bohannan

CONTENTS

1 Justification of Variance Partitioning **1**

2 Additive Variances under Pairwise Interactions **2**

3 Tables of Parameter Values used in Simulations **5**

1 | JUSTIFICATION OF VARIANCE PARTITIONING

Here we provide calculations to justify the partitioning of variance introduced in section 2.1 of the main text. The calculations here hold for any collection of numerically coded factors $f = (f_1, \dots, f_n)^\dagger$. In the main text, the f_i are equal to the allele counts g_i for $i = 1, \dots, L$, and are equal to relative microbial abundances m_i for $i = L+1, \dots, L+S$. Instead of the additive effect specific to genotype-microbiome pairs α_{gm}, we focus on obtaining an expression for the additive effect for the vector of arbitrary factors f, denoted α_f. The additivity constraint on α_f implies $\alpha_f = \alpha_0 + \sum_i \alpha_i f_i$, which we abbreviate to $\alpha_f = \alpha_0 + \langle \alpha, f \rangle$ with $\alpha = (\alpha_1, \dots, \alpha_n)^\dagger$ the vector of additive effects associated with each component of f, and α_0 is the intercept of the linear model. In the main text $\alpha_i = \gamma_i$ for $i = 1, \dots, L$ and $\alpha_i = \omega_i$ for $i = L+1, \dots, L+S$. We make the decomposition $z_f - \bar{z} = \delta_f = \alpha_f + \rho_f$, i.e., ρ is the residual. Following the definition of P_R in the main text we have $P_R = \sum_f p_f \rho_f^2$, where p_f is the frequency of f in the population. Then P_R can be rewritten as $P_R = \sum_f p_f (\delta_f - \alpha_f)^2$. Our goal is to minimize P_R. Partial differentiation with respect to the intercept returns

$$\partial_{\alpha_0} P_R = -2 \sum_f p_f (\delta_f - \alpha_0 - \langle \alpha, f \rangle) = -2 \sum_f p_f \rho_f = -2\bar{\rho}. \tag{1}$$

The optimality condition $\partial_{\alpha_0} P_R = 0$ then implies $\bar{\rho} = 0$, and hence we can set $\alpha_0 = -\sum_f p_f \langle \alpha, f \rangle = -\langle \alpha, \bar{f} \rangle = -\sum_i \alpha_i \bar{f}_i$. Partial differentiation with respect to additive effect α_i for $i = 1, \dots, n$ returns

$$\partial_{\alpha_i} P_R = -2 \sum_f p_f (\delta_f - \alpha_0 - \langle \alpha, f \rangle) f_i = -2 \sum_f p_f \rho_f f_i = -2 \operatorname{Cov}(\rho, f_i), \tag{2}$$

where the last equality holds because $\bar{\rho} = 0$. Then $\partial_{\alpha_i} P_R = 0$ implies $\operatorname{Cov}(\rho, f_i) = 0$. The expression for $\partial_{\alpha_i} P_R$ can also be written as

$$\partial_{\alpha_i} P_R = -2 \operatorname{Cov}(\delta_f, f_i) + 2 \operatorname{Cov}(\langle \alpha, f \rangle, f_i), \tag{3}$$

Note that $\operatorname{Cov}(\delta_f, f_i) = \operatorname{Cov}(z_f, f_i)$ and $\operatorname{Cov}(\langle \alpha, f \rangle, f_i) = \sum_j \alpha_j \operatorname{Cov}(f_j, f_i)$. Hence, $\partial_{\alpha_i} P_R = 0$ also implies

$$\operatorname{Cov}(z_f, f_i) = \sum_j \alpha_j \operatorname{Cov}(f_i, f_j). \tag{4}$$

Writing $\operatorname{Cov}(z_f, f)$ as the vector with ith entry $\operatorname{Cov}(z_f, f_i)$ and Σ as the $n \times n$ matrix with i,jth entry $\operatorname{Cov}(f_i, f_j)$, the expression above can be written in vector-matrix notation as $\operatorname{Cov}(z_f, f) = \Sigma\, \alpha$. Hence, $\alpha = \Sigma^{-1} \operatorname{Cov}(z_f, f)$ when Σ^{-1} exists and $\alpha_0 = -\langle \alpha, \bar{f} \rangle$ with $\bar{f} = (\bar{f}_1, \dots, \bar{f}_n)^\dagger$. This establishes the definition of additive genetic and microbial effects provided in the main text when Σ is non-singular.

The result $\operatorname{Cov}(\rho_f, f_i) = 0$ implies $\sum_f p_f \alpha_f \rho_f = 0$, which then formally establishes the partitioning of the variance of the marginal value $\operatorname{Var}(z_f) = \sum_f p_f (z_f - \bar{z})^2$ as $\operatorname{Var}(z_f) = P_A + P_R$, with $P_A = \sum_f p_f \alpha_f^2$. More generally, given K different classes of factors such that $f = (f_1^1, \dots, f_{n_1}^1, \dots, f_1^K, \dots, f_{n_K}^K)^\dagger$ and $\alpha = (\alpha_1^1, \dots, \alpha_{n_1}^1, \dots, \alpha_1^K, \dots, \alpha_{n_K}^K)^\dagger$, the expression for P_A can be rewritten as

$$P_A = \sum_{k=1}^{K} P_{A,k} + \sum_{k=1}^{K} \sum_{\ell=1}^{k-1} C_{A,k,\ell}, \tag{5}$$

where $P_{A,k} = \sum_f p_f \alpha_{f^k}^2$ and $C_{A,k,\ell} = 2\sum_f p_f \alpha_{f^k}\alpha_{f^\ell}$, with $f^k = (f_1^k, \dots, f_{n_k}^k)^\dagger$, $\alpha^k = (\alpha_1^k, \dots, \alpha_{n_k}^k)^\dagger$, and $\alpha_{f_k} = \alpha_0^k + \langle \alpha^k, f^k \rangle$. Here we have introduced new partition-specific intercepts, which must satisfy $\alpha_0 = \sum_k \alpha_0^k$, and so we can take $\alpha_0^k = -\langle \alpha^k, \bar{f}^k \rangle$. With this choice the contributions to α_f from each partition are centered, so the resulting partition of variance comes out in terms of variances and covariances (without this choice of α_0^k we would have sums of squares instead of (co)variances). This returns our partitionings introduced in section 2.1 and in section 2.3.2 as special cases. Finally, our choice for α_0^k implies

$$P_{A,k} = \sum_{i=1}^{n_K} (\alpha_i^k)^2 \operatorname{Var}(f_i^k) + 2\sum_{i=1}^{n_k}\sum_{j=1}^{i-1} \alpha_i^k \alpha_j^k \operatorname{Cov}(f_i^k, f_j^k), \tag{6a}$$

$$C_{A,k,\ell} = 2\sum_{i=1}^{n_k}\sum_{j=1}^{n_\ell} \alpha_i^k \alpha_j^\ell \operatorname{Cov}(f_i^k, f_j^\ell), \tag{6b}$$

and this returns equations (5) of the main text as a special case.

The above partitioning of P_A is unique only when Σ is non-singular. When Σ is singular, the Moore–Penrose pseudoinverse of Σ is commonly used in place of Σ^{-1}. This approach is useful for approximating P_A, but the estimate of α loses biological significance as a consequence of its lack of uniqueness.

2 | ADDITIVE VARIANCES UNDER PAIRWISE INTERACTIONS

Here we compute expressions for host trait variation using a model that assumes genotypic-microbic values are determined by mechanistic additive effects of host allele counts $g_1, \dots, g_L$ and microbial abundances $m_1, \dots, m_S$ in addition to mechanistic effects explained by products of host allele counts $g_i g_j$, products of microbial abundances $m_i m_j$, and products of host allele counts and microbial abundances $g_i m_j$. This model incorporates all possible pairwise products of trait mediating factors analyzed, and therefore generalizes the model presented in section 5 of the main text which focused only on products of host allele counts and microbial abundances. The model here can be expressed as

$$z_{gm} = z_0 + \sum_{i=1}^{L} \mathring{\gamma}_i g_i + \sum_{i=1}^{L}\sum_{j=1}^{i} \mathring{\gamma}_{ij} g_i g_j + \sum_{i=1}^{S} \mathring{\omega}_i m_i + \sum_{i=1}^{S}\sum_{j=1}^{i} \mathring{\omega}_{ij} m_i m_j + \sum_{i=1}^{L}\sum_{j=1}^{S} \mathring{\chi}_{ij} g_i m_j. \tag{7}$$

Our goal here is to compute $G_A = \sum_{gm} p_{gm}\gamma_g^2$, $M_A = \sum_{gm} p_{gm}\omega_g^2$, and $C_A = \sum_{gm} p_{gm}\gamma_g\omega_m$ given the above model under the assumption that all factors $g_1, \dots, g_L, m_1, \dots, m_S$ are linearly independent so that Σ^{-1} exists (which implies uniqueness of G_A, M_A, C_A). To begin, note that

$$\begin{aligned}\operatorname{Cov}(z_{gm}, g_k) = &\sum_{i=1}^{L} \mathring{\gamma}_i \operatorname{Cov}(g_i, g_k) + \sum_{i=1}^{S} \mathring{\omega}_i \operatorname{Cov}(m_i, g_k) \\ &+ \sum_{i=1}^{L}\sum_{j=1}^{i} \mathring{\gamma}_{ij} \operatorname{Cov}(g_i g_j, g_k) + \sum_{i=1}^{S}\sum_{j=1}^{S} \mathring{\omega}_{ij} \operatorname{Cov}(m_i m_j, g_k) + \sum_{i=1}^{L}\sum_{j=1}^{S} \mathring{\chi}_{ij} \operatorname{Cov}(g_i m_j, g_k),\end{aligned} \tag{8a}$$

$$\mathrm{Cov}(z_{gm}, m_k) = \sum_{i=1}^{L} \mathring{\gamma}_i \mathrm{Cov}(g_i, m_k) + \sum_{i=1}^{S} \mathring{\omega}_i \mathrm{Cov}(m_i, m_k) + \sum_{i=1}^{L}\sum_{j=1}^{i} \mathring{\gamma}_{ij} \mathrm{Cov}(g_i g_j, m_k) + \sum_{i=1}^{S}\sum_{j=1}^{i} \mathring{\omega}_{ij} \mathrm{Cov}(m_i m_j, m_k) + \sum_{i=1}^{L}\sum_{j=1}^{S} \mathring{\chi}_{ij} \mathrm{Cov}(g_i m_j, m_k). \tag{8b}$$

Writing $\mathring{\alpha} = (\mathring{\gamma}_1, \dots, \mathring{\gamma}_L, \mathring{\omega}_1, \dots, \mathring{\omega}_S)$, and v the vector-valued function of g and m with kth entry

$$v_k = \sum_{i=1}^{L}\sum_{j=1}^{i} \mathring{\gamma}_{ij} \mathrm{Cov}(g_i g_j, g_k) + \sum_{i=1}^{S}\sum_{j=1}^{i} \mathring{\omega}_{ij} \mathrm{Cov}(m_i m_j, g_k) + \sum_{i=1}^{L}\sum_{j=1}^{S} \mathring{\chi}_{ij} \mathrm{Cov}(g_i m_j, g_k), \quad k = 1, \dots, L, \tag{9a}$$

$$v_k = \sum_{i=1}^{L}\sum_{j=1}^{i} \mathring{\gamma}_{ij} \mathrm{Cov}(g_i g_j, m_{k-L}) + \sum_{i=1}^{S}\sum_{j=1}^{i} \mathring{\omega}_{ij} \mathrm{Cov}(m_i m_j, m_{k-L}) + \sum_{i=1}^{L}\sum_{j=1}^{S} \mathring{\chi}_{ij} \mathrm{Cov}(g_i m_j, m_{k-L}), \quad k = L+1, \dots, L+S, \tag{9b}$$

we have $\mathrm{Cov}(z_{gm}, a) = \Sigma\mathring{\alpha} + v$. Hence $\alpha = \mathring{\alpha} + \Sigma^{-1} v$, and writing $u = \Sigma^{-1} v$ the vector-valued function of g and m resulting from a transformation on v, we have $\gamma_i = \mathring{\gamma}_i + u_i$ and $\omega_i = \mathring{\omega}_i + u_{i+L}$.

$$\gamma_g = \sum_{i=1}^{L} (\mathring{\gamma}_i + u_i) g_i, \quad \omega_g = \sum_{i=1}^{S} (\mathring{\omega}_i + u_{i+L}) m_i. \tag{10a}$$

Consequently

$$\bar{\gamma} = \sum_{i=1}^{L} \mathring{\gamma}_i \bar{g}_i + \overline{g_i u_i}, \quad \bar{\omega} = \sum_{i=1}^{S} \mathring{\omega}_i \bar{m}_i + \overline{m_i u_{i+L}}, \tag{11}$$

where $\overline{g_i u_i}$ and $\overline{m_i u_{i+L}}$ are averages of $g_i u_i$ and $m_i u_{i+L}$ across genotype-microbiome pairs in the host population. To simplify expressions we write $[x] = x - \bar{x}$ and $[xy] = xy - \overline{xy}$. This leads to

$$[\gamma_g]^2 = \sum_{i=1}^{L} \mathring{\gamma}_i^2 [g_i]^2 + 2\mathring{\gamma}_i [g_i][g_i u_i] + [g_i u_i]^2 + 2\sum_{i=1}^{L}\sum_{j=1}^{i-1} \mathring{\gamma}_i \mathring{\gamma}_j [g_i][g_j] + \mathring{\gamma}_i [g_i][g_j u_j] + \mathring{\gamma}_j [g_j][g_i u_i] + [g_i u_i][g_j u_j], \tag{12a}$$

$$[\omega_m]^2 = \sum_{i=1}^{S} \mathring{\omega}_i^2 [m_i]^2 + 2\mathring{\omega}_i [m_i][m_i u_{i+L}] + [m_i u_{i+L}]^2 + 2\sum_{i=1}^{S}\sum_{j=1}^{i-1} \mathring{\omega}_i \mathring{\omega}_j [m_i][m_j] + \mathring{\omega}_i [m_i][m_j u_{j+L}] + \mathring{\omega}_j [m_j][m_i u_{i+L}] + [m_i u_{i+L}][m_j u_{j+L}] \tag{12b}$$

$$[\gamma_g][\omega_m] = \sum_{i=1}^{L}\sum_{j=1}^{S} \mathring{\gamma}_i \mathring{\omega}_j [g_i][m_i] + \mathring{\gamma}_i [g_i][m_j u_{j+L}] + \mathring{\omega}_j [m_j][g_i u_i] + [g_i u_i][m_j u_{j+L}]. \tag{12c}$$

We can therefore write

$$G_A = \sum_{i=1}^{L} \mathring{\gamma}_i^2 \,\mathrm{Var}(g_i) + 2\mathring{\gamma}_i \,\mathrm{Cov}(g_i, g_i u_i) + \mathrm{Var}(g_i u_i) + 2\sum_{i=1}^{L}\sum_{j=1}^{i-1} \mathring{\gamma}_i \mathring{\gamma}_j \,\mathrm{Cov}(g_i, g_j) + \mathring{\gamma}_i \,\mathrm{Cov}(g_i, g_j u_j) + \mathring{\gamma}_j \,\mathrm{Cov}(g_j, g_i u_i) + \mathrm{Cov}(g_i u_i, g_j u_j), \tag{13a}$$

$$M_A = \sum_{i=1}^{S} \mathring{\omega}_i^2 \operatorname{Var}(m_i) + 2\mathring{\omega}_i \operatorname{Cov}(m_i, m_i u_{i+L}) + \operatorname{Var}(m_i u_{i+L})$$
$$+ 2\sum_{i=1}^{S}\sum_{j=1}^{i-1} \mathring{\omega}_i \mathring{\omega}_j \operatorname{Cov}(m_i, m_j) + \mathring{\omega}_i \operatorname{Cov}(m_i, m_j u_{j+L}) + \mathring{\omega}_j \operatorname{Cov}(m_j, m_i u_{i+L}) + \operatorname{Cov}(m_i u_{i+L}, m_j u_{j+L}), \quad (13b)$$

$$C_A = 2\sum_{i=1}^{L}\sum_{j=1}^{S} \mathring{\gamma}_i \mathring{\omega}_j \operatorname{Cov}(g_i, m_i) + \mathring{\gamma}_i \operatorname{Cov}(g_i, m_j u_{j+L}) + \mathring{\omega}_j \operatorname{Cov}(m_j, g_i u_i) + \operatorname{Cov}(g_i u_i, m_j u_{j+L}). \quad (13c)$$

3 | TABLES OF PARAMETER VALUES USED IN SIMULATIONS

TABLE 1 Parameter values used for simulating data presented in Figure 5 of main text

Symbol	Meaning	Value
nr	Number of times to initialize parental population	100
sr	Number of times to repeat selection of hosts	20
or	Number of times to repeat production of offspring	20
h^2	Expected proportion of additive genetic variance	0.25
λ^2	Expected proportion of additive lineal variance	0.25
ν^2	Expected proportion of additive non-lineal variance	0.25
ε^2	Expected proportion of additive external variance	0.25
L	Number of host genetic loci	100
SL	Number of lineal microbe taxa	100
SN	Number of non-lineal microbe taxa	100
SE	Number of external microbe taxa	100
$\mathbb{E}P$	Expected total phenotypic variance	100
p	Probability of alternate allele at each locus in parental genomes	0.5
n	Size of host populations	1000
s	Selection coefficient	0.001
KL	Expected abundance for lineal microbe taxa	50
KN	Expected abundance for non-lineal microbe taxa	50
KE	Expected abundance for external microbe taxa	50

TABLE 2 Parameter values used for simulating data presented in Figures 3 and 4 of main text

Symbol	Meaning	Value
T	Number of host generations	10
ts_rep	Number of time series to repeat	20
nr	Number of times to initialize parental population	50
sr	Number of times to repeat selection of hosts	10
or	Number of times to repeat production of offspring	10
h^2_{GLNE}	Expected proportion of additive genetic variance for GLNE	0.25
λ^2_{GLNE}	Expected proportion of additive lineal variance for GLNE	0.25
v^2_{GLNE}	Expected proportion of additive non-lineal variance for GLNE	0.25
$\varepsilon^2 GLNE$	Expected proportion of additive external variance for GLNE	0.25
h^2_{GLN}	Expected proportion of additive genetic variance for GLN	0.33
λ^2_{GLN}	Expected proportion of additive lineal variance for GLN	0.33
v^2_{GLN}	Expected proportion of additive non-lineal variance for GLN	0.33
ε^2_{GLN}	Expected proportion of additive external variance for GLN	1e-3
h^2_{GL}	Expected proportion of additive genetic variance for GL	0.5
λ^2_{GL}	Expected proportion of additive lineal variance for GL	0.5
v^2_{GL}	Expected proportion of additive non-lineal variance for GL	1e-3
ε^2_{GL}	Expected proportion of additive external variance for GL	1e-3
h^2_G	Expected proportion of additive genetic variance for G	0.99
λ^2_G	Expected proportion of additive lineal variance for G	1e-3
v^2_G	Expected proportion of additive non-lineal variance for G	1e-3
ε^2_G	Expected proportion of additive external variance for G	1e-3
L	Number of host genetic loci	100
SL	Number of lineal microbe taxa	100
SN	Number of non-lineal microbe taxa	100
SE	Number of external microbe taxa	100
$\mathbb{E}[P_{GLNE}]$	Expected total phenotypic variance for GLNE	100
$\mathbb{E}[P_{GLN}]$	Expected total phenotypic variance for GLN	75
$\mathbb{E}[P_{GL}]$	Expected total phenotypic variance for GL	50
$\mathbb{E}[P_G]$	Expected total phenotypic variance for G	25
p	Probability of alternate allele at each locus in parental genomes	0.5
n	Size of host populations	1000
s	Selection coefficient	0.001
KL	Expected abundance for lineal microbe taxa	50
KN	Expected abundance for non-lineal microbe taxa	50
KE	Expected abundance for external microbe taxa	50
